# Hydrogel Electrolytes Based on Xanthan Gum: Green Route towards Stable Dye-Sensitized Solar Cells

**DOI:** 10.3390/nano10081585

**Published:** 2020-08-12

**Authors:** Simone Galliano, Federico Bella, Matteo Bonomo, Guido Viscardi, Claudio Gerbaldi, Gerrit Boschloo, Claudia Barolo

**Affiliations:** 1 Department of Chemistry and NIS Interdepartmental Center and INSTM Reference Centre, University of Torino, Via Pietro Giuria 7, 10125 Torino, Italy; simone.galliano@unito.it (S.G.); matteo.bonomo@unito.it (M.B.); guido.viscardi@unito.it (G.V.); 2 GAME Lab, Department of Applied Science and Technology (DISAT), Politecnico di Torino, Corso Duca degli Abruzzi 24, 10129 Torino, Italy; federico.bella@polito.it (F.B.); claudio.gerbaldi@polito.it (C.G.); 3 Department of Chemistry, Uppsala University, Box 523, 75120 Uppsala, Sweden; 4 ICxT Interdepartmental Center, Università degli Studi di Torino, Lungo Dora Siena 100, 10153 Torino, Italy

**Keywords:** aqueous dye-sensitized solar cells, xanthan gum, design of experiments, stability, biosourced polymer

## Abstract

The investigation of innovative electrolytes based on nontoxic and nonflammable solvents is an up-to-date, intriguing challenge to push forward the environmental sustainability of dye-sensitized solar cells (DSSCs). Water is one of the best choices, thus 100% aqueous electrolytes are proposed in this work, which are gelled with xanthan gum. This well-known biosourced polymer matrix is able to form stable and easily processable hydrogel electrolytes based on the iodide/triiodide redox couple. An experimental strategy, also supported by the multivariate chemometric approach, is used here to study the main factors influencing DSSCs efficiency and stability, leading to an optimized system able to improve its efficiency by 20% even after a 1200 h aging test, and reaching an overall performance superior to 2.7%. In-depth photoelectrochemical investigation demonstrates that DSSCs performance based on hydrogel electrolytes depends on many factors (e.g., dipping conditions, redox mediator concentrations, etc.), that must be carefully quantified and correlated in order to optimize these hydrogels. Photovoltaic performances are also extremely reproducible and stable in an open cell filled in air atmosphere, noticeably without any vacuum treatments.

## 1. Introduction

Nowadays, sustainable solar energy harvesting technologies able to provide enhanced performance under low or weak irradiation (e.g., on a cloudy day, indoor, etc.) and possessing aesthetically pleasing/functional features, suitable for building integration (BIPV), are highly suited as complementary renewable sources to Si-based photovoltaics from a smart-grid energy perspective [1,2,3,4]. Among them, dye-sensitized solar cells (DSSCs) are one of the most interesting choices [5,6,7]. However, many issues remain to be solved before the widespread commercialization of practical DSSCs, safety as well as long-term stability being among the main drawbacks [8]. Actually, high-efficiency DSSCs are prepared mainly with organic solvent-based liquid electrolytes, such as acetonitrile (ACN) and methoxypropionitrile (MPN). These are often oil derivatives characterized by toxicity and flammability; in addition, they possess high vapor pressure and volatility, which often straightforwardly lead to electrolyte leakage and decrease of photovoltaic performance over time [9,10].

Bearing this in mind and with the idea of creating efficient, safe and low-cost DSSCs, the research moved towards alternative solvents [11,12,13]. Above all, water-based DSSCs are amongst the best solutions, providing reduced costs, nonflammability, good stability and environmental compatibility [14]. In recent years, scientific literature on this topic has significantly increased [15,16,17,18,19]. Devices have been reported, which achieve efficiency close to 6% with a mixed water/organic electrolyte [20], or even 5.5% and 7% with 100% aqueous electrolytes based on a cobalt [21] and iodine/iodide [22] redox couple, respectively. The latter results have been obtained with an optimized photoanode and an innovative PEDOT (poly(3,4-ethylenedioxythiophene))-derivative based counter electrode.

Even more interestingly, the possibility of trapping the liquid solvent in a solid or quasi-solid matrix can reduce electrolyte leakage, correspondingly increasing long-term stability [10,23,24]: polymer networks are highly effective in this respect, due to their easy synthesis and tunable properties. However, the use of a hydrogel is much less explored in the literature. Camacho et al. employed a K-carrageenan hydrophilic polymer as a gelling agent in pure water, but the overall performance was lower than 0.01% [25]. More recently, Saraubh and coworkers [26] reported on the use of a saturated chenodeoxycholic acid (CDCA) liquid electrolyte that was then jellified with 62% w/w block copolymer (EO_52_-PO_35_-EO_52_), resulting in photoconversion efficiency (PCE) ≈ 1%. The same authors employed a slightly different block copolymer, viz. Pluronic F77 (PEO_53_-PPO_34_-PEO_53_) in an aqueous solution containing 0.5 M LiI/0.05 M I_2_/0.2 M tert-butyl pyridine (TBP) with 45% w/w polymer to solution composition leading to efficiency close to 2% [27]. Zhang et al. employed an inorganic templating agent (SiO_2_) to build up a jellified electrolyte (efficiency = 1.48%) [28]. Interesting results (up to 4%) [29] have also been obtained with high voltage redox couples; however, the use of cobalt complexes still undermines the overall sustainability. Moreover, water-based quasi-solid electrolytes ensuring the highest efficiencies are usually prepared with a large quantity of synthetic macromolecules derived from the petrochemical industry, which actually makes the DSSC technology not 100% renewable or eco-friendly [30,31,32].

**Figure 1:**
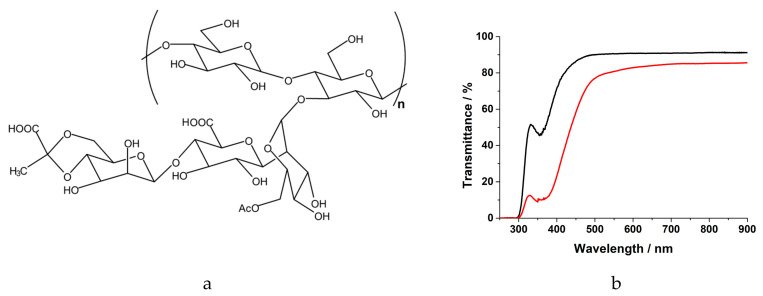
(**a**) General molecular structure of XG, with “n” ranging between 2000 and 20,000. (**b**) UV−Vis transmittance spectra of aqueous liquid (black) and xanthan-based hydrogel (red) electrolytes.

Bearing in mind the idea to investigate the feasibility of stable, but sustainable solar cells with safe and truly nontoxic components, we decided to focus our efforts on low-cost, abundant bio-derived polymers and critical raw material (CRM)-free redox couples as attractive and feasible alternatives to common organic electrolyte redox mediators [33,34,35,36,37,38,39]. In this context, we employed a standard Pt-based counter-electrode, but a further improvement in the sustainability of aqueous DSSCs should consider the development of alternative counter-electrodes [40,41,42]. Following our preliminary results on the use of carboxymethyl cellulose (CMC) as a biopolymer matrix for aqueous electrolytes [34], here we propose a different bioderived polymer, namely the xanthan gum (XG). XG is a low-cost, water-soluble polysaccharide secreted by *Xanthomonas campestris* bacterium and commonly used in the food and cosmetic industries as a thickening and stabilizing agent [43]. Its primary structure is based on a pentasaccharide repeating unit, comprising glucose, mannose and glucuronic acids (Figure 1a) [43], able to form a three-dimensional hydrogel network with a thixotropic property [44]. With a specific view towards DSSC scalability, this property should allow the XG-based hydrogel to effectively penetrate into the mesopores of the TiO_2_ photoanode as a liquid does, thus overcoming the pore filling issues of common highly viscous polymer-based electrolytes [30,45]. XG has been investigated as a polymer matrix in DSSC electrolytes by Park et al., who actually developed a hydrogel-like system containing 50% of organic solvent (e.g., MPN) [46]. The efficiency of the “quasi” hydrogel electrolyte was lower compared to its liquid counterpart, but it nonetheless showed a noticeable long-term stability over 288 days of the aging test.

Inspired by this interesting result, here we propose the first example of 100% truly aqueous electrolytes gelled with biosourced XG. The first part is focused on the comparison between our standard aqueous liquid electrolyte (previously optimized thanks to the introduction of CDCA [19] and TiCl_4_ treatment of photoelectrodes [47]) with the related XG-based hydrogel, discussing their photovoltaic performances and cell properties. Then, we present a multivariate chemometric experimental strategy (design of experiments, DoE) [48] on hydrogel electrolytes to simultaneously evaluate how the conditions of anode sensitization and electrolyte composition could affect DSSCs performance and long-term stability. Although this approach is scarcely exploited in the photovoltaic field, DoE can considerably improve the final information about the investigated system, as demonstrated in the present work [49,50].

## 2. Materials and Methods

Sodium iodide (NaI), iodine (I_2_), xanthan gum (XG, *M*_w_ = 4–12 × 10^6^ g mol^−1^), chenodeoxycholic acid (CDCA), ethanol (EtOH), acetone (Ac), t-butanol (t-BuOH), titanium tetrachloride (TiCl_4_), hexachloroplatinic acid (H_2_PtCl_6_) and acetonitrile (ACN) were purchased from Merck KGaA. Deionized water (DI-H_2_O, 18 MΩ cm at 25 °C) was obtained with a Direct-Q 3 UV Water Purification System (Millipore). The sensitizing dye 2-[{4-[4-(2,2-diphenylethenyl)phenyl]-1,2,3,3a,4,8b-hexahydrocyclopento[b]indole-7-yl}methylidene]- cyanoacetic acid (D131) was purchased from Inabata Europe S.A. All the reagents were purchased at the highest degree of purity available and they were employed without further purification. Fluorine-doped tin oxide (FTO) glass plates (sheet resistance 15 Ω sq^−1^, purchased from Solaronix) were cut into 2 cm × 1.5 cm sheets and used as substrates for the fabrication of both photoanodes and counter electrodes.

The aqueous liquid electrolyte was prepared as follows: a homogeneous supersaturated solution was obtained by adding excess CDCA in DI-H_2_O and stirring overnight at 40 °C; it was then cooled to room temperature and filtered with paper to obtain a clear solution into which NaI and I_2_ were then dissolved by stirring for 2 h. The concentration of NaI and I_2_ ranged from 1 to 5 M and from 10 to 30 mM, respectively.

Hydrogel electrolytes were prepared by simply stirring XG powder (3 or 5 wt.%) into the already prepared aqueous electrolytes (as explained before) for 2 h at 40 °C. All electrolytes were stored in closed vials in the dark and at room temperature.

FTO covered glasses were rinsed in mixed Ac/EtOH in an ultrasonic bath for 10 min; solvent traces were removed by compressed air. Photoanodes with transparent mesoporous TiO_2_ (≈6 μm thickness and 0.25 cm^2^ active area) were prepared by screen-printing a layer of TiO_2_ paste (18NR-T, Dyesol) onto the conductive side, followed by a stepwise thermal treatment at increasing temperatures (ramp 10 °C min^−1^): 15 min at 25, 125 and 375 °C, respectively, and, finally, 20 min at 480 °C. Photoanodes were then dipped into a solution of TiCl_4_ (40 mM in DI-H_2_O) for 30 min at 70 °C. TiCl_4_ treatment was proved to improve the photocurrent and efficiency of aqueous solar cells [47]. After rinsing with DI-H_2_O and EtOH, photoanodes were heated up to 520 °C for 20 min and then cooled down to 50 °C and soaked (still hot) in the dye solution, which was composed of D131 dye (0.5 mM) and CDCA, as coadsorbents in different ratios (between 1:18 and 1:50 dye to CDCA), in a mixture of ACN/t-BuOH 1:1. The dipping step into dye solutions was carried out at 25 (±1) °C for 5 h under dark conditions and shaking in a Büchi Syncore platform. After dye loading, photoanodes were washed in Ac to remove residual dye, and dried with compressed air.

With regard to the preparation of counter electrodes, FTO glasses were platinized by spreading a drop of H_2_PtCl_6_ (5.0 mM solution in isopropanol) onto the conductive side and heating up to 450 °C for 30 min.

For the liquid electrolyte-based devices, the photoanode and counter electrode were firstly assembled under a hot press (30 s at 90 °C) with a Surlyn thermoplastic gasket (internal area 0.6 cm × 0.6 cm) as spacer (≈ 50 μm thickness), taking care of the overlapping of the active areas. Then, the aqueous liquid electrolyte was injected by vacuum process through a hole in the Surlyn frame, which was then sealed by commercial epoxy glue. Differently, hydrogel electrolyte (~2 mg) was spread onto the TiO_2_ layer with a spatula; the photoanode and counter electrode were then sealed with a Surlyn thermoplastic gasket by hot pressing. It is worth mentioning that the XG-based electrolyte is not sticking. After fabrication, the sealed DSSCs were stored simply on laboratory countertops and aged at ambient temperature.

Diffusion coefficients were calculated by means of both diffusion limiting current and electrochemical impedance spectroscopy as reported in the literature [51]. Current−voltage (J−V) characteristics were measured under 1 sun light intensity (100 mW cm^−2^, AM 1.5G) provided by an AAA class sun simulator equipped with a 150 W xenon arc lamp (91195A, Newport Corp., Irvine, CA, USA) and connected to a digital source meter (2420, Keithley Instrument Inc., Cleveland, OH, USA), after calibration with a silicon reference solar cell (91150V, Newport Corp.). Three consecutive J−V measurements were performed on solar cells and their performance was monitored over time. Incident-photon-to-current conversion efficiency (IPCE) spectra were recorded using a combined computer-controlled setup consisting of a xenon light source (Spectral Products ASB-XE-175) and a monochromator (Spectral Products CM110) connected to a digital source meter (2700, Keithley Instrument Inc.), which was calibrated using the same certified reference solar cell mentioned above. The dependence of current and voltage as a function of irradiation, electron lifetime and transport time, and extracted charge measurements were performed by a toolbox (DN-AE01, Dyenamo, Stockholm, Sweden) with a white light-emitting diode (Natural white S42182H 1W, Seoul Semiconductors, Ansan-si, Korea) as the light source [7]. Briefly, the electron lifetimes and transports were calculated by monitoring photocurrent and photovoltage rise transients at different light intensities upon applying a small square wave modulation to the base light intensity. Extracted charge measurements were performed by illuminating the cell under open-circuit (Qoc) or short-circuit (Qsc) conditions and then turning the lamp off. Then, the current was measured for a certain period and integrated to obtain the extracted charge [52,53,54]. The term “toolbox” indicates different photoelectrochemical techniques useful to investigate the properties of DSSCs under operating conditions [55].

## 3. Results and Discussion

### 3.1. Aqueous Liquid vs. XG-Based Hydrogel Electrolytes

#### 3.1.1. Electrolyte Characterization

Aqueous liquid and hydrogel electrolytes were compared in terms of photovoltaic performance and studied by means of toolbox measurements. Photoanode sensitization conditions (CDCA:dye molar ratio equal to 18:1) and liquid electrolyte (LIQ-1) formulation (iodide and iodine concentrations at 5 M and 30 mM, respectively, in a CDCA supersaturated solution) were selected following our previous results on aqueous DSSCs [47]. Being commonly the limiting redox species, especially in quasi-solid and solid electrolytes [56], a sufficiently high amount of I_2_ should have avoided carrier mobility and diffusion issues, at the same time improving long-term stability of solar cells.

Hydrogel electrolyte (XG-1) was prepared by adding 5 wt.% of xanthan gum to the liquid counterpart. In accordance with our previous experiments on CMC-based hydrogel electrolytes, here we found that the amount of 5 wt.% XG produced a homogeneous viscous gel, which was consistent and self-standing even when overturning the bottle [34]. In Figure 1b both liquid and hydrogel electrolytes are shown, together with the corresponding UV−Vis absorption spectra. Hydrogel electrolytes appeared much darker than their liquid counterpart. Interestingly, the darkening disappeared during the hot-press sealing and slowly appeared again while the cell cooled down to ambient temperature, finally stabilizing to the initial color after few hours. This phenomenon, in addition to the blue-greenish shades, suggests a possible “starch effect” due to the interactions between triiodide in the electrolyte and the polymer [37]. Further investigation of this effect will be discussed in a forthcoming paper. It is worth mentioning that the pH of aqueous-based electrolytes could deeply influence the photoelectrochemical performance of the device. Therefore, we measured the pH of LIQ-1 throughout the preparation procedure: deionized water was 7.05; quite unexpectedly, the pH of the CDCA-saturated solution did not change too much, but it slightly decreased to 6.33. The addition of NaI and I_2_ simply led to a partial basification of the solution (pH = 6.95). Concerning XG-1, measuring the pH was not possible due to the pH meter instability during the measurement in the gel phase. Yet, we can reasonably assume that the addition of XG (3 wt.%) did not modify consistently the pH of the solution. Indeed, the pH of an aqueous solution of xanthan gum (3 wt.%) is close to neutrality (i.e., 6.98). To further confirm that, we also measured the equilibrium potential of both LIQ-1 and XG-1 samples, and they were equal to 270 and 262 mV vs. Ag/AgCl, respectively. Straightforwardly, the redox potential of the electrolyte seems to be independent of the presence of the XG matrix.

#### 3.1.2. Photoelectrochemical and Stability Characterization

**Figure 2:**
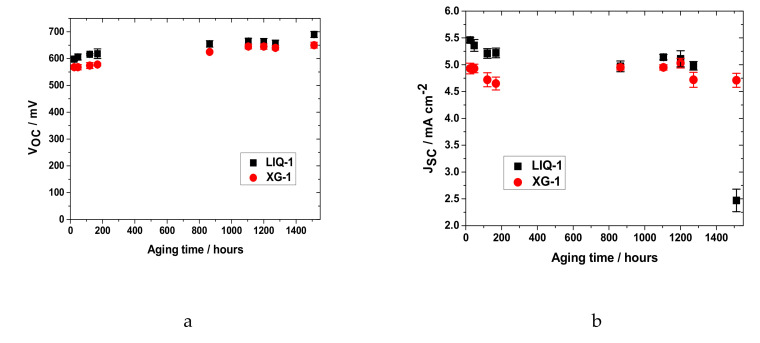

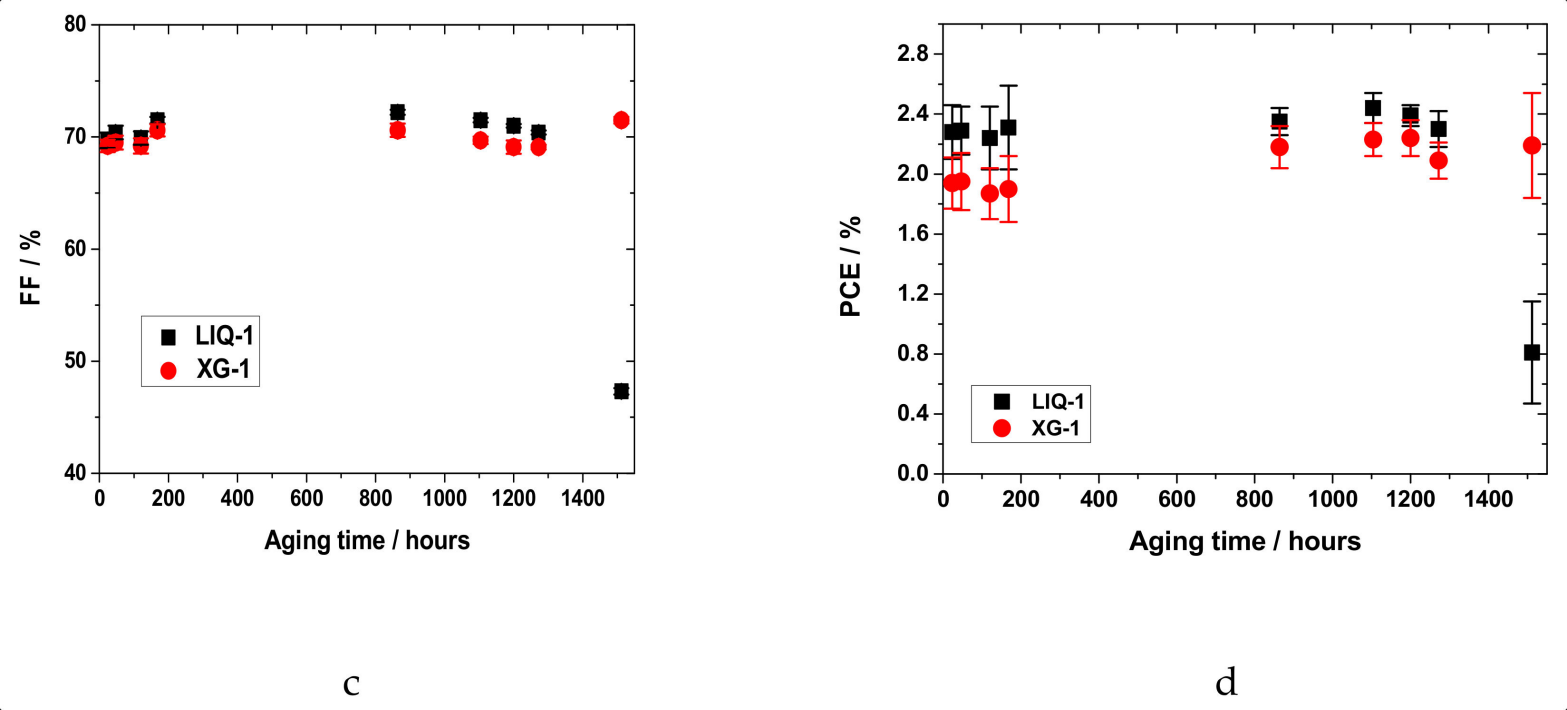
Best photovoltaic performance (**a**) V_OC_ (voltage at open circuit); (**b**) J_SC_ (short circuit current); (**c**) FF (fill factor); (**d**) PCE (power conversion efficiency); and relative errors over time for aqueous liquid (black squares) and hydrogel (red circles) dye-sensitized solar cells (DSSCs). Data points refer to the third of three consecutive measurements at 1 sun.

The photovoltaic performance of DSSCs filled with aqueous liquid and hydrogel electrolytes were measured under standard 1 sun irradiation and monitored for over two months (≈1500 h) of aging at ambient laboratory temperature. Two cells were prepared for each electrolyte and the photovoltaic parameters of the best cell are shown over time in Figure 2.

Surprisingly, both electrolytes showed very similar photovoltaic results. In the first hours of aging, the efficiencies of liquid and gel DSSCs achieved values of 2.28 and 1.93%, respectively. The lower performance (−15%) of the hydrogel is likely ascribed to little drops in both V_OC_ (−5%) and J_SC_ (−10%). To exclude a mass-limitation effect caused by the presence of XG matrix we calculated the diffusion coefficient of the limiting species (i.e., triiodide) of both LIQ-1 and XG-1 from the polarization curve [57]. XG-1 showed a slightly lower coefficient (=1.3 × 10^−6^ cm^2^ s^−1^) compared to its liquid counterpart (=2.6 × 10^−6^ cm^2^ s^−1^), ascribable to the higher viscosity throughout the polymeric matrix. Notwithstanding, the limiting current density (J_lim_) of XG-1 was at least three times higher than the one produced by the device (i.e., 15 mA cm^−2^). This assured that limitations in ion diffusion could not reduce the overall current of the device.

**Figure 3:**
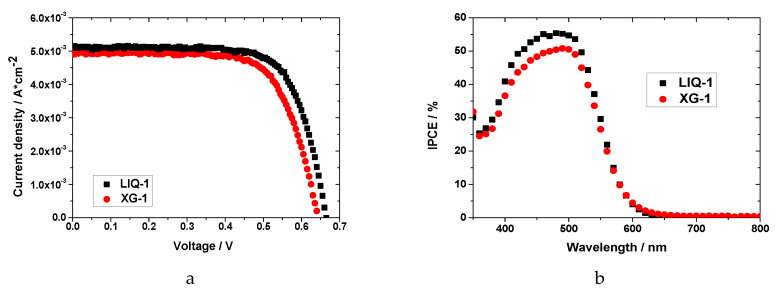
(**a**) J−V curves and (**b**) IPCE spectra for the best LIQ-1 (black squares) and XG-1 (liquid circles) DSSCs measured after ≈1100 h of aging.

Very interestingly, the efficiencies of both electrolytes slowly increased upon aging: both the cells achieved the highest performances after ≈1100 h of aging time (Table 1 and Figure 3). Remarkably, the efficiency gap between LIQ-1 and XG-1 decreased over time; this was due to a J_SC_ loss in the liquid cell, which affected the overall efficiency. In particular, a slight decrease in the photocurrent was found to be constant throughout the aging test and became quite drastic after ≈1250 h, knocking down the efficiency to below 1%. The sudden drop in PCE is due to the sealing failure and subsequent electrolyte leakage. It should be pointed out that, during aging, the channel used to introduce the liquid electrolyte could be a “preferred” path for solvent leakage, jeopardizing the lifetime of LIQ-1. On the other hand, hydrogel-based cells exhibited impressive stability in all their parameters, which even increased after more than 1500 h of the aging test. Indeed, the presence of jellified electrolyte prevented leakage from the cell by “freezing” it in the XG matrix.

The difference in the photocurrent between LIQ-1 and XG-1 was well-evidenced by the IPCE measurements, as shown in Figure 3b (best cell after 3 days). The spectra showed an enlarged peak with the maximum photoconversion around 480 nm, consistent with the absorbance of D131 dye onto the TiO_2_ surface [58]. As expected, the LIQ-1 cell exhibited a slightly higher value (~55%) than XG-1 (~51%), in line with the higher photogenerated current. No other significant differences were observed in the IPCE spectra of the two cells.

These results indicate that the XG-based matrix slightly negatively influenced the photocurrent because of an expected matrix-effect, but did not affect the photo-responsive profile of the solar cells, in spite of the darker aspect of the cells when compared to the liquid counterparts (vide supra). However, a sort of light-filter effect, due to the permeation of dark electrolyte into TiO_2_ pores, cannot be excluded.

#### 3.1.3. Toolbox Analyses

Aqueous liquid and hydrogel electrolytes were also studied in lab-scale DSSCs by means of toolbox techniques to shed light on their different performances. Dependence of V_OC_ and J_SC_ on light, electron lifetime and electron transport time were studied under different light intensities.

**Figure 4:**
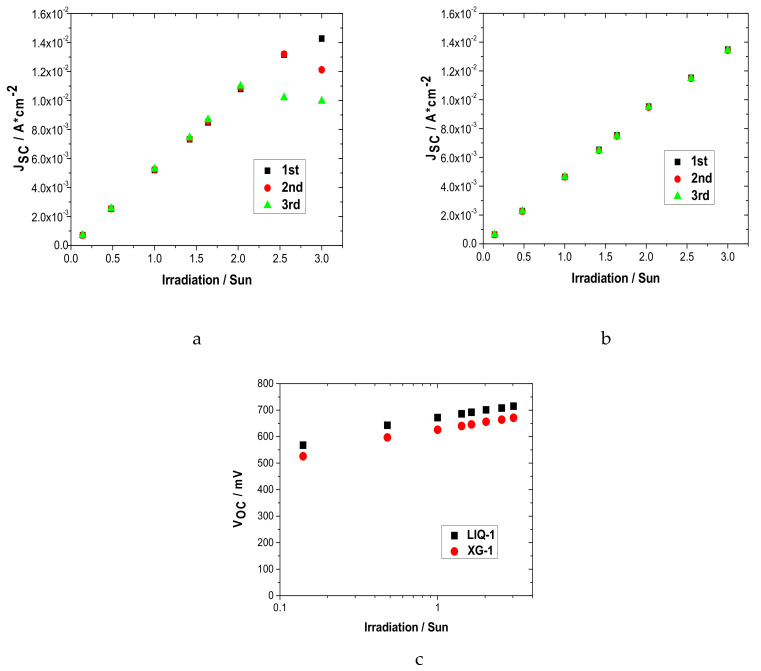
Photocurrent (**a**) LIQ-1 and (**b**) XG-1 based DSSCs, and (**c**) photovoltage in function of the light intensity for the best DSSCs, performed after ≈800 h of aging test.

The J_SC_ and V_OC_ parameters in function of the light intensity (up to 3 suns) are shown in Figure 4.

As previously observed, XG-1 based cells exhibited lower photocurrent than LIQ-1 up to 3 suns (13.5 vs. 14.3 mA cm^−2^, respectively, Figure 4). However, it showed an almost perfectly linear dependence (R^2^ = 0.999) on the light intensity. The differences in light dependence became evident under high intensity (>2 sun) with the number of performed measurements (i.e., three different cycles on the same device): LIQ-1 showed a remarkable decrease in J_SC_, while XG-1 was perfectly stable. This behavior is likely related to a possible fastening of the recombination processes that occur during prolonged operation under strong irradiation levels. On the other hand, the presence of the XG matrix could partially protect the excessive irradiation of the redox mediator. The inefficient photocurrent generation of the liquid electrolyte appears clearer when monitoring the J_SC_ continuously while keeping the irradiation constant (Figure S1). This behavior was found to be consistent within a set of five (namely identical) devices.

As soon as the illumination was turned on, the LIQ-1 based cell exhibited higher photocurrent than the hydrogel one (5.2 vs. 5.0 mA cm^−2^, respectively) and remained stable for over 40 s of continuous working. The slightly lower J_SC_ is ascribable to the higher absorbance of XG-based electrolyte that partially reduces the amount of radiation reaching the photoanode. It is worth mentioning that this effect makes just a modest contribution to the reduction of the current density, being the device illuminated from the photoanode side. Indeed, the filter effect produced by XG-1 becomes meaningful just for the inner portion of the photoanode. After 40 s, J_SC_ remarkably decreased for the liquid cell (−12%), stabilizing again after 20 s, then keeping its value constant (4.6 mA cm^−2^). Differently, photocurrent generation of the XG-1 based cell was stable during the experiment, even slightly increasing, thus definitely outperforming (+9%) the liquid electrolyte after 50 s of continuous operation. This could be ascribed to the protective effect of the XG matrix. We hypothesize that XG could prevent the excessive irradiation and the straightforward heating of the electrolyte. As a matter of fact, under continuous operation iodide species could be subjected to photodegradation reactions leading to a modification in the concentration of the active species in the electrolyte. This explanation is consistent with the reported results, with an initial stabilization period in which the photocurrent slightly increases. Then, LIQ-1 suffered a small (but limited in time and magnitude) performance depletion that led to a stabilization of the photocurrent after 60 s. Conversely, XG-based cells exhibited very stable J_SC_ values.

Overall, electrolyte bleaching under light was not detected (coherently with our previous studies on aqueous DSSCs), thus we did not observe the disappearance of I_3_^−^ ions found by Macht et al. through photocurrent imaging techniques [59]. Thus, it seems that the iodine-based redox shuttle kept in an aqueous environment and exposed to 1 sun visible irradiation is rather stable (even in the presence of XG) and relevant modifications would occur only under more stressful conditions, i.e., exposure to O_3_ atmosphere [60].

Concerning photovoltage values (Figure 4), both cells exhibited a similar dependence on light irradiation, with a slope of ≈90 mV per decade with increasing intensity. This value is much higher than the ideal 59 mV, which is likely ascribable to some limitations of the water-based system, regardless of the polymeric matrix.

**Figure 5:**
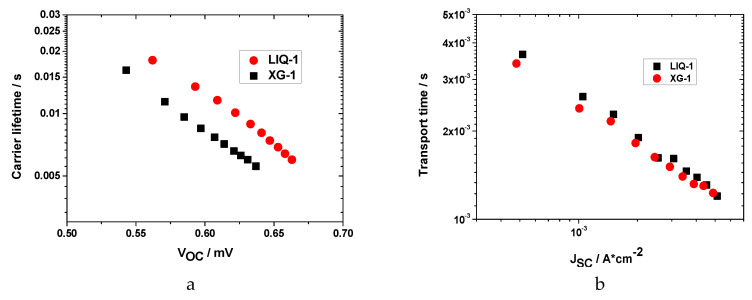

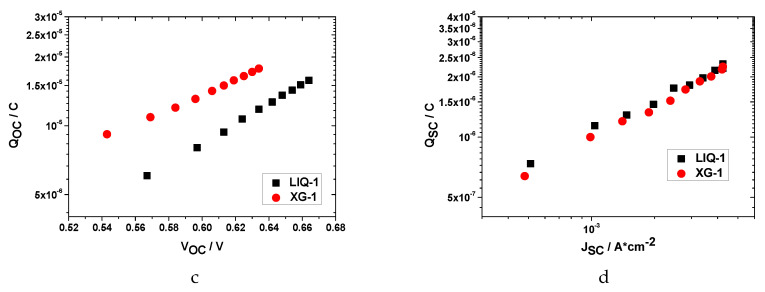
(**a**) Electron lifetime and (**b**) transport time. Extracted charges for aqueous liquid and hydrogel cells as a function of (**c**) V_OC_ and (**d**) J_SC_ under different light intensities.

Figure 5a shows electron lifetime as a function of V_OC_ under different light intensities for both liquid and hydrogel cells. The electron lifetimes are lower than the typical values of organic solvent-based electrolytes, showing that recombination processes affect the water-based system. Moreover, the liquid electrolyte exhibited a slightly longer lifetime compared to the hydrogel one, but their values were quite close, indicating that XG does not significantly increase the electron recombination processes, as could be expected.

Similar conclusions can be derived by analyzing the electron transport time shown in Figure 5b. However, even if electron lifetime and transport time were measured under different cell conditions, the latter was only ≈3−5 fold lower than lifetime, indicating that recombination likely occurs before the electron is collected.

Moreover, the extracted charge from TiO_2_ in open-circuit conditions was found to be lower than typical values of organic solvent-based cells (Figure 5c). However, as expected, the charge increased with V_OC_, in accordance with an exponential distribution of trapping states below the conduction band energy, E_CB_. In such a test, a higher amount of charge was extracted in the presence of the hydrogel electrolyte. The higher values could be attributed to a shift of E_CB_ (toward the redox potential of the mediator), if no changes occurred in trap density [61]. The trap density was constant with different electrolytes, which was supported by charge extraction measurements under short-circuit conditions (Figure 5d), where no significant differences were observed between cells. Hence, the conduction band edge may be shifted by the interaction with XG polymer, as was also proved by the lower V_OC_ generated in the presence of the jellified electrolyte. Additionally, we likely exclude a change in the redox potential of the electrolyte due to the use of XG matrix (as already discussed above). It should be pointed out that Mott–Schottky (MS) plot is often employed in the determination of flat band potential of semiconductors [62]. Unfortunately, MS is highly reliable just for flat (or slightly porous electrodes [63]). In the present case, the highly porous nature of our TiO_2_ electrodes make the MS approach unsuitable. Furthermore, in this context, the aim was mainly to evidence some differences between LIQ and XG more than make an accurate determination of the Fermi’s level of TiO_2_ electrodes.

In conclusion, liquid and hydrogel electrolytes exhibited similar behavior under operating conditions, which indicates that the XG matrix does not detrimentally affect the photo-electrochemical processes occurring in the DSSC. Lower V_OC_ and J_SC_ parameters, in addition to the short electron lifetime, could be ascribed to a slight increase in electron recombination processes. Moreover, XG seems to positively shift the TiO_2_ conduction band edge, probably as consequence of the adsorption of positive ions released by the polymer (i.e., protonation). Above all, the most interesting result is the relative stability enhancement achieved by the electrolyte gelation with XG polymer, which candidates it among the most promising low-cost and bioderived electrolyte jellifying agents for DSSCs.

### 3.2. A Multivariate Investigation of XG-Based Hydrogel Electrolytes

#### 3.2.1. Experimental Design

To deepen the knowledge of XG-based hydrogel electrolytes and to improve the overall photovoltaic performances, a DoE was planned and performed on a large series of hydrogel-based lab-scale DSSCs. DoE enables the simultaneous evaluation of the effect of different factors, while performing a limited number of experiments. However, DSSCs possess an intrinsic high variability, which limits the possible observable variations due to a controlled factor. Hence, the DoE was planned as a simple screening of three relevant factors, while maintaining constant as much as possible all the other not observed variables. In order to carry out a multivariate experimental design and the related statistical evaluation, the software MODDE (version 11.0.2.2309, Umetrics) was used.

In this study, concentrations of redox mediators [I_2_ (x_1_) and NaI (x_2_)] in the electrolyte and the CDCA:dye molar ratio (x_3_) adopted for anode sensitization were selected as the three relevant factors. In fact, XG-based matrix could interact with redox carriers leading to reduced carrier mobility, carrier complexation, or even other effects. Hence, evaluating different amounts, and ratios, of the two redox mediators should allow us to understand their effect on performance and to identify the best trade-off to achieve higher efficiency with better stability. Indeed, CDCA is typically used as co-adsorbent in dye solution to reduce aggregation of dye molecules onto the TiO_2_ surface, thus improving the dye coverage of the photoanode. It has been recently demonstrated that interdye hole transport can occur in a monolayer of highly packed D131 molecules, leading to an increase in recombination processes [64]. Hence, different CDCA amounts could also lead to changes in cell photovoltaic performances.

The effects of these three factors were evaluated by a thoroughly detailed analysis of the cells photovoltaic performance and characteristics (V_OC_, J_SC_, FF, PCE and long-term stability). In order to evaluate only these experimental factors, all the other variables related to cell fabrication were kept constant.

Hydrogel electrolytes consisted of NaI (1 to 5 M) and I_2_ (10 to 30 mM) in CDCA-saturated water with 3 wt.% of XG. In this case, no significant darkening of the cell occurred, probably due to the lower amount of XG added into the electrolytes. Stable hydrogels were obtained also in this case. Instead, the CDCA:dye molar ratio in the sensitization solution ranged from 18 to 50 (9 to 25 mM of CDCA). A two-level full factorial design was planned, the list of experiments is summarized in Table 2.

Table 2 also lists the photovoltaic parameters measured under 1 sun standard irradiation after ≈100 h from cell sealing. It was observed that cell performances significantly increased during the first hours from device sealing, indicating a sort of “activation period” required for these hydrogel-based solar cells. As a result, performing measurements in this initial period can lead to wrong conclusions, as also mentioned by some authors [65]. The measured efficiencies ranged from 1.57 (cell 5) to 2.55% (cell 8), and a detailed analysis of the experimental data was properly carried out to understand the reasons for the observed performance variations.

Analysis of the experimental data as a function of the controlled factors allowed us to obtain a mathematical model describing the effect of each factor and their possible interactions on the cell photovoltaic performance. The model was fitted by means of PLS regression [66]. Hence, all four responses were fitted simultaneously, giving an overview of how the experimental factors affected all of the measured responses. The summary of fit (R^2^, model validity and reproducibility) for each response is shown in Figure S2. The high values of R^2^ indicate that each model explains well the measured experimental variation as a function of changed factors. Reproducibility and model validity values showed that experimental data were not particularly affected by pure error, and the latter was not significantly different from model error.

The effect of factors and their interactions, which all together represent the model coefficients, were studied on each photovoltaic response, and are plotted in Figure S3.

#### 3.2.2. Efficiency vs. Experimental Factors

**Figure 6:**
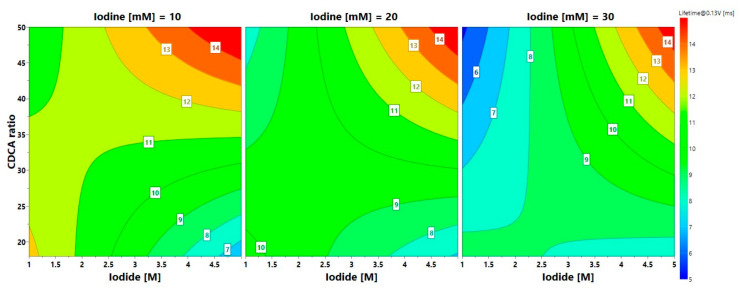
Isoresponse contour plots of electron lifetime as a function of different experimental factors.

The CDCA:dye ratio exhibited an overall positive effect on photovoltaic performance, increasing both V_OC_ and J_SC_ (Figure S3). Generally, the presence of this co-adsorbent in the sensitizing bath improves charge injection in TiO_2_, by reducing aggregation and stacking between dye molecules. These phenomena decrease electron injection and increase the charge recombination processes. This effect could become more evident in the presence of increased amounts of oxidized species in the electrolyte (free I_2_ and I_3_^−^). Moreover, CDCA can improve the photoanode wettability, being less hydrophobic than D131 dye, and as a result, photocurrent and voltage increase. Besides this, we also considered the linear interactions (CDCA × I^−^): it gave a positive effect, suggesting that CDCA exhibits different effects depending on the iodide ions amount. At lower [I^−^], CDCA did not significantly affect either voltage or current, while at higher [I^−^] it showed a remarkably positive effect. The effect could also be observed on electron lifetime values for cells 6 and 8, as shown in Figure 6.

A shift in I_2_/I_3_^−^ equilibrium toward triiodide, due to the increase in the concentration of iodide ions, and a better dye coverage due to CDCA-promoted effect, led to reduced recombination and longer lifetimes [61]. This evidence was different from some of the literature reports [67], and was likely ascribed to lower dye concentration levels adopted by other groups, leading to an insufficient/inhomogeneous coverage of the electrode surface. Even a shift in TiO_2_ levels could occur due to a reduced amount of dye molecules on the anode surface. As regards the interaction CDCA × I_2_, it seemed not to particularly affect the cells performance, but only FF values changed.

Positive variations of iodine concentration tended to increase the overall efficiency, in particular due to the higher J_SC_ and FF (Figure S3). This improvement could be related to the increased occurrence of charge transfer phenomena. Charge extraction measurements at open-circuit condition showed lower values with a low amount of I_2_ (Figure S4). In addition, longer electron lifetime and transport time values were observed by lowering [I_2_] (Figure S5). On the contrary, no significant, or scarcely negative, effects were observed on V_OC_ due to [I_2_] variation. This latter fact suggested that, even if I_2_ should have increased V_OC_ due to a redox potential shift, it could detrimentally affect the photovoltage by enhancing the amount of recombining species (i.e., I_3_^−^). In Figure S4, the extracted charge at short-circuit condition showed similar values for all cells, while it was significantly lower only in the case of high [I_2_] and low [I^−^]. This evidence seemed to confirm that recombination was the main cause of voltage loss.

As far as iodide concentration is concerned, the effect on J_SC_ and PCE was generally positive, while it was the opposite for V_OC_ and FF values. In particular, voltage decreased due to the shift of redox potential toward higher energy. Moreover, the positive interactions with CDCA and I_2_ likely suggested improved wettability and reduced recombination, and this was confirmed by the generally higher electron lifetime values (Figure 6).

The extracted charge at open circuit was also barely lower with high [I^−^] (Figure S4), which might be ascribed to a negative shift of the TiO_2_ conduction band and/or a fewer number of trap states. Moreover, considering that the Q_OC_ equation contains a term to take into account the charge accumulated at the electrode/electrolyte interfaces, changes in double-layer capacitance and Stark effect may also be important factors [68]. On the other hand, FF was negatively affected likely because of electron transfer issues at the cathode/electrolyte interface, where the large amount of iodide ions increased the resistance, especially at low I_3_^−^ concentration. On the contrary, photocurrent was remarkably increased by iodide amount; as mentioned before, it could be due to the reduced surface tension of the electrolyte and improved wettability. Moreover, the increase in [I^−^] corresponded to an increase also in the amount of Na^+^ ions, which could adsorb on TiO_2_ surface and shift the conduction band edge as in the well-known case of lithium ions [69]. The shift caused opposite effect on V_OC_ and J_SC_ values.

#### 3.2.3. Stability vs. Experimental Factors

**Figure 7:**
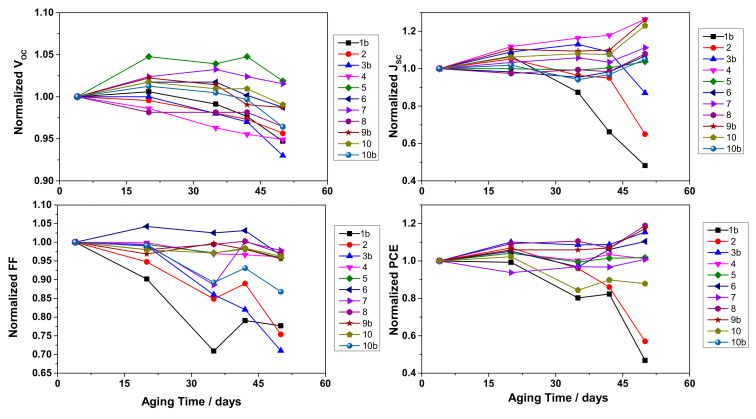
Photovoltaic parameters of DoE cells during the 50 days of the aging test. Data points refer to the third of three consecutive measurements at 1 sun. Lines just connect data points and have no physical meaning.

Finally, the long-term stability of lab-scale cells was also evaluated by monitoring their photovoltaic performances over a period of 50 days (1200 h), as shown in Figure 7. In general, aqueous hydrogel electrolyte exhibited an impressively stable efficiency, showing similar parameters, even higher, during the aging period for almost all the cells. Surprisingly, the photocurrent increased over time continuously, while only three cells showed remarkable J_SC_ losses.

**Figure 8:**
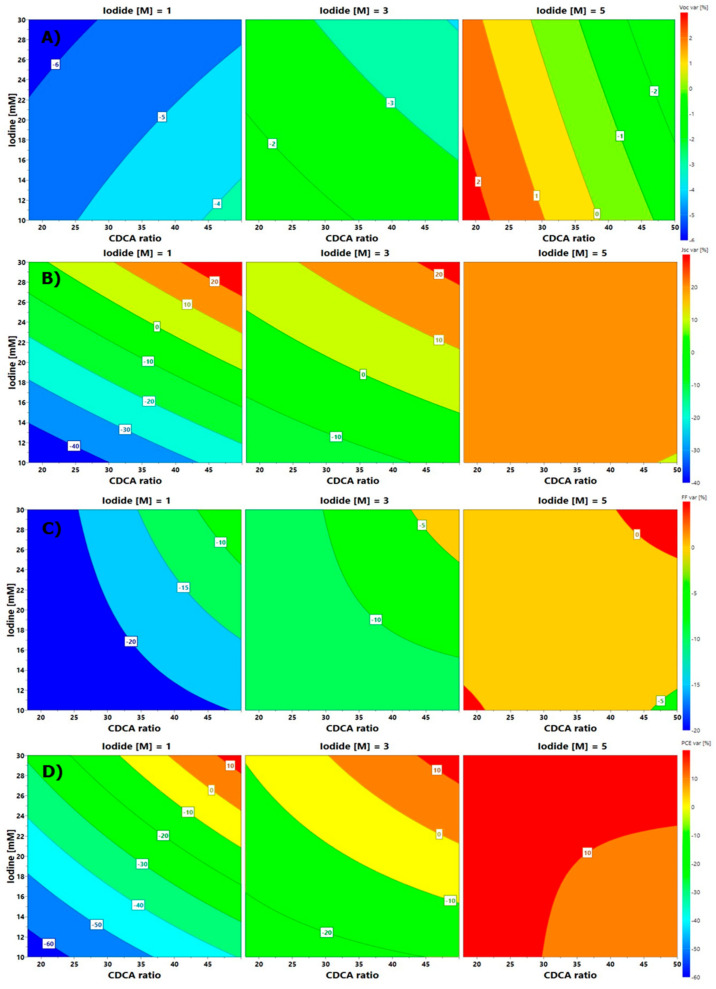
Contour plots for (**A**) V_OC_, (**B**) J_SC_, (**C**) FF and (**D**) PCE variation as a function of factors.

A multivariate analysis was performed to evaluate the aging of the cells; however, in this case the experimental variability was very high, which did not allow us to consider as significant many of the model coefficients. Nevertheless, DoE analysis was effective to observe some trends in the stability test due to applied variations in experimental factors. Figure 8 shows the isoresponse plots for each photovoltaic parameter calculated by the model. Above all, higher iodide concentrations facilitated longer stability, as observable comparing the three graphs for each photovoltaic parameter. Concerning CDCA:dye ratio, it seemed to reduce the V_OC_ stability, but surprisingly the other photovoltaic performances were greatly enhanced, demonstrating a negligible desorption of dye molecules from TiO_2_ surface upon aging. The best combination of all factors may be obtained around the central point of the experimental domain, where the most significant cell stability was recorded, which was provided by cell 9b: it exhibited the best stability, with an outstanding +20% in its PCE after 1200 h, leading to an efficiency equal to 2.75% (V_OC_ = 623 mV, J_SC_ = 6.58 mA cm^−2^, FF = 67%).

The proper combination of experimental and characterization strategies led to an optimized lab-scale DSSC reaching an overall performance equal to 2.75%, actually reproducible and very stable upon aging, which, to the best of our knowledge, is the most remarkable result obtained so far with a truly aqueous electrolyte based on a biosourced polymer, iodine/iodide redox couple and CDCA additive.

## 4. Conclusions

A number of severe challenges is nowadays pushing forward the development of DSSCs with enhanced environmental sustainability. The use of water as the sole electrolyte solvent is amongst the most intriguing choices for the purpose. In this work, 100% water-based hydrogel electrolytes based on biosourced XG were successfully proposed as alternative, cheap, green electrolytes for DSSCs, where toxic and volatile organic solvents as well as oil-derived polymers and CRMs are fully avoided. Newly developed materials and devices were investigated by means of in-depth electrochemical analyses; a specific and tailored multivariate approach was also included. Actually, thorough DoE analysis allowed the clarification of some correlations between the chosen experimental factors (i.e., dipping conditions and redox mediator concentrations) and the final photovoltaic performances and stability of lab-scale devices.

It is well-known that critical issues in aqueous systems chiefly include the higher recombination processes and the lower mobility of ionic charges. Here we demonstrated that the electrolyte jellification by the use of XG did not significantly worsen these parameters. Overall, some optimization is needed, particularly in terms of long-term stability of the devices; however, thanks to the properly optimized experimental conditions achieved through DoE, we were able to effectively reduce leakage of iodine out of the device. It allowed a +20% improvement of efficiency after the 1200 h aging test, which led to an overall PCE of 2.75% (higher for hydrogel with respect to its liquid counterpart). Performances were found to be reproducible and stable in an open cell filled in air atmosphere, without any time/energy consuming vacuum treatments. These preliminary results, obtained on transparent devices, readily assembled in lab-scale configuration with yellow dye and standard counter electrode, confirm that hydrogel electrolytes based on a sustainable biosourced polymer offer remarkable opportunities to further develop low-cost, sustainable aqueous based DSSCs, which provide the same performance as the corresponding liquid electrolyte counterpart, but with remarkably higher stability. Future strategies, which are already under investigation in our laboratories, will include the replacement of platinum by cheap and abundant alternatives, such as biosourced carbonaceous materials, the use of natural dyes and the development of fully solid-state electrolytes by quick and reliable preparation procedures.

## Figures and Tables

**Table 1:** Best photovoltaic performances for aqueous liquid and hydrogel DSSCs measured after ≈1100 h of aging.

Electrolyte	V_OC_ (mV)	J_SC_ (mA cm^−2^)	FF (%)	PCE (%)
LIQ-1	665	5.14	71.5	2.44
XG-1	638	4.95	71.3	2.23

**Table 2:** DoE matrix consisting of 11 experiments, with their experimental conditions (x_1_, x_2_ and x_3_) and measured photovoltaic parameters (J_SC_, V_OC_, FF and PCE). Data refer to the third of three consecutive measurements at 1 sun, performed after ≈100 h from device sealing. Two nominally identical cells were used in each experiment, therefore 22 lab-scale DSSCs were assembled for this study. The best cell for each of the 11 experimental conditions was then fully characterized through photovoltaic measurements and toolbox techniques.

Cell Name	I_2_ (x_1_, mM)	NaI (x_2_, M)	CDCA:Dye Ratio (x_3_)	V_oc_ (mV)	J_sc_ (mA cm^−2^)	FF (%)	PCE (%)
1b	10	1	18	681	3.49	71.2	1.69
2	10	1	50	668	3.65	70.6	1.72
3b	30	1	18	658	4.37	71.1	2.05
4	30	1	50	660	4.32	70.5	2.01
5	10	5	18	587	4.00	67.0	1.57
6	10	5	50	634	5.18	64.8	2.13
7	30	5	18	586	4.81	68.5	1.93
8	30	5	50	647	5.56	70.8	2.55
9b	20	3	34	631	5.22	70.1	2.31
10	20	3	34	634	5.13	69.8	2.27
11	20	3	34	642	4.78	68	2.09

## Supplementary Materials

The following are available online at https://www.mdpi.com/2079-4991/10/8/1585/s1, Figure S1: Device aging under 1 sun; Figure S2: DoE parameters; Figure S3: DoE coefficient significance; Figure S4: Extracted charges; Figure S5: Carrier lifetime and transport time.
