# Supplementary material for: Hydrogel Electrolytes Based on Xanthan Gum: Green Route towards Stable Dye-Sensitized Solar Cells
Source: Nanomaterials (Basel). 2020 Aug 12;10(8):1585. doi: 10.3390/nano10081585 (PMC7466386; doi:10.3390/nano10081585)
Supplement: Supplementary file 1 [file nanomaterials-10-01585-s001.pdf]

Supporting information

Hydrogel Electrolytes Based on Xanthan Gum: Green Route towards Stable Dye-Sensitized Solar Cells

Simone Galliano , Federico Bella, Matteo Bonomo, Guido Viscardi, Claudio Gerbaldi, Gerrit Boschloo * and Claudia Barolo *

* Correspondence: claudia.barolo@unito.it (C.B.); gerrit.boschloo@kemi.uu.se (G.B.)

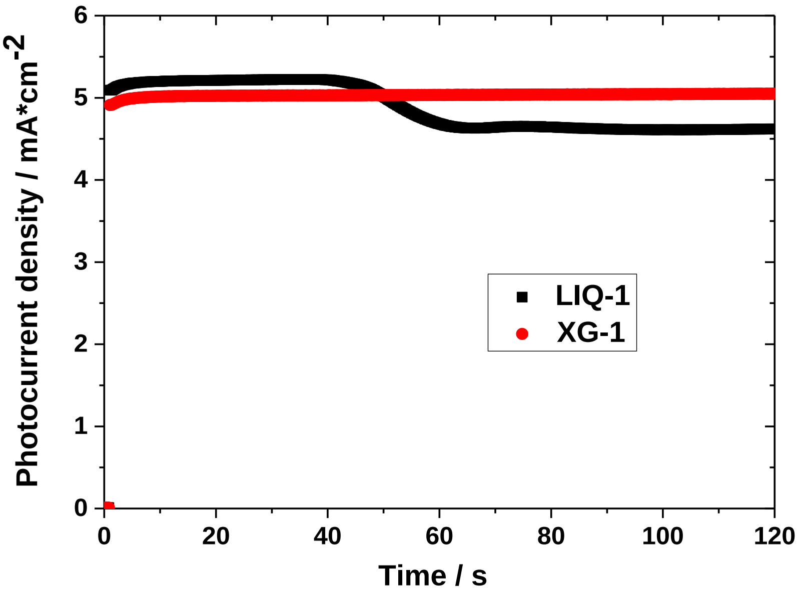

Figure S1. Short-circuit photocurrent density of liquid- and hydrogel-based cells measured under 1 sun irradiation.

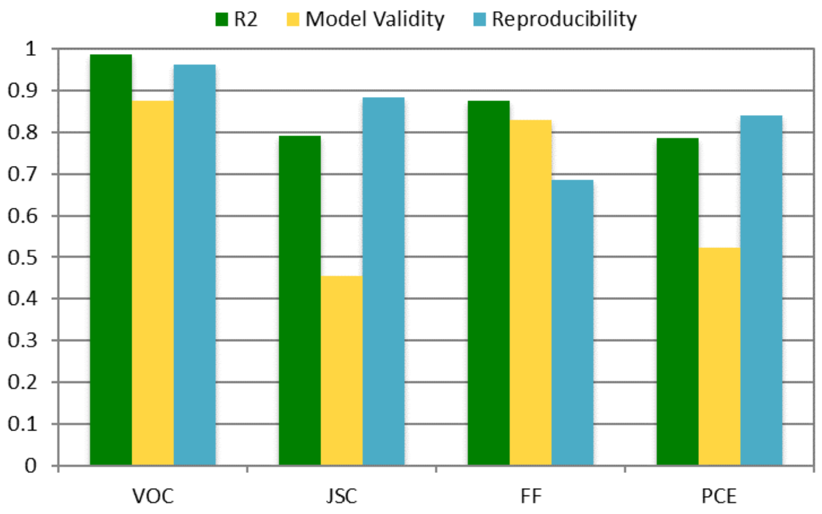

Figure S2. Summary of fit for each response showing R^2 value, model validity and reproducibility.

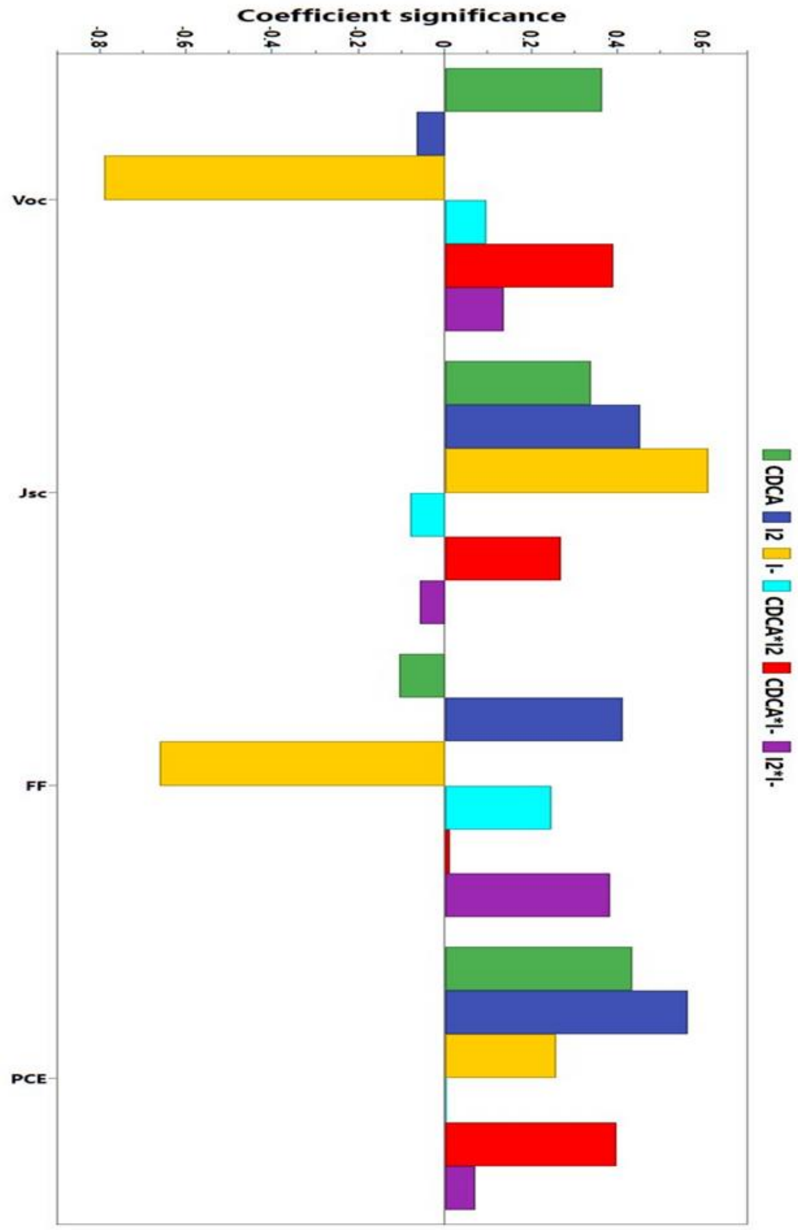

Figure S3. Coefficient significance of model terms on selected photovoltaic responses (J_{SC}, V_{OC}, FF PCE).

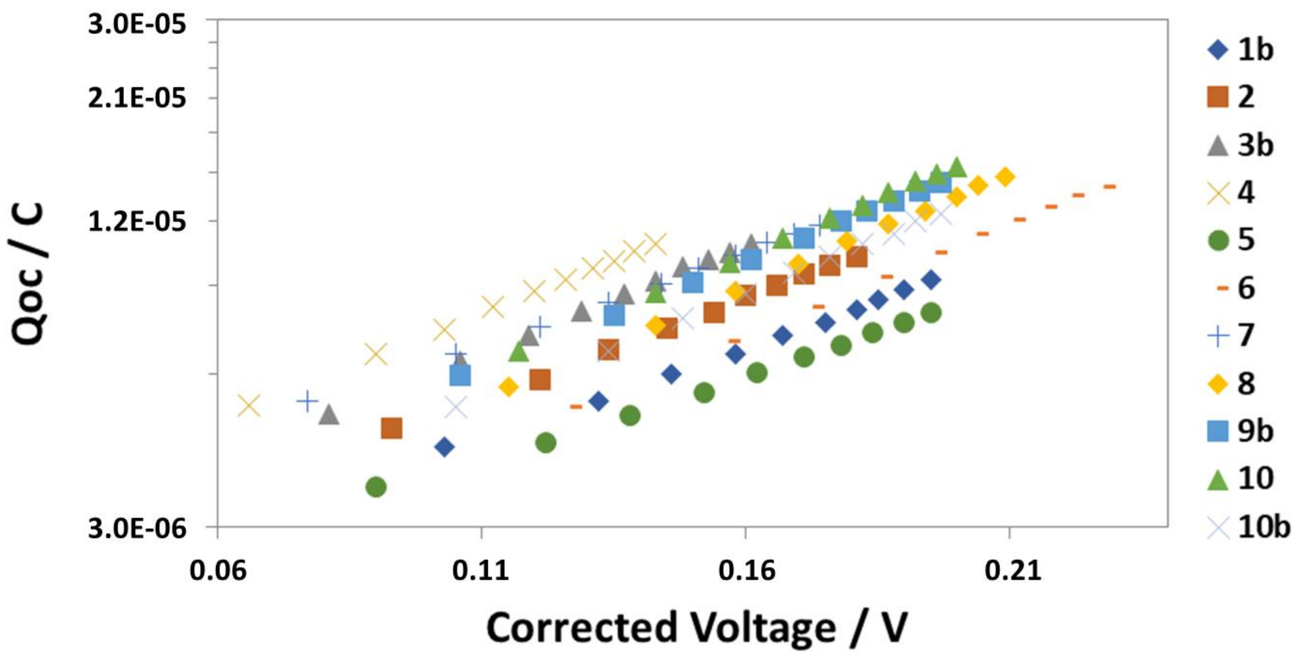

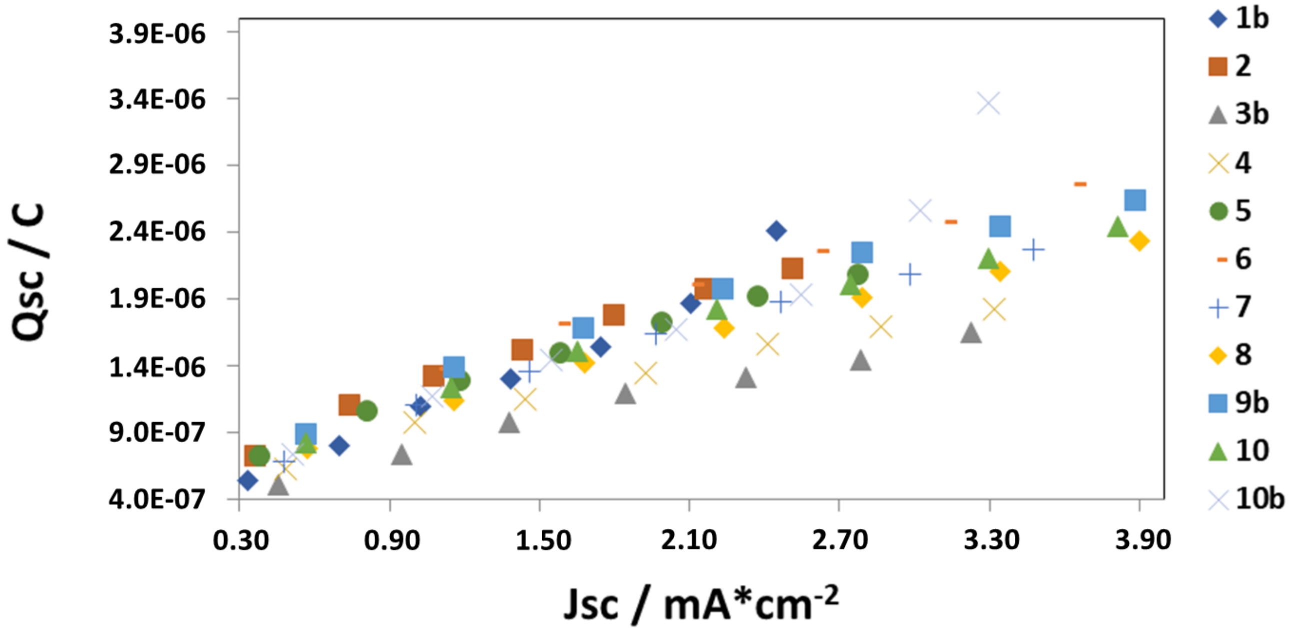

Figure S4. Extracted charges for DoE cells as a function of V_{OC} (up) and J_{SC} (down) under different light intensities. V_{OC} was corrected considering the shift of $E_{f,redox}$ due to the different redox concentrations.

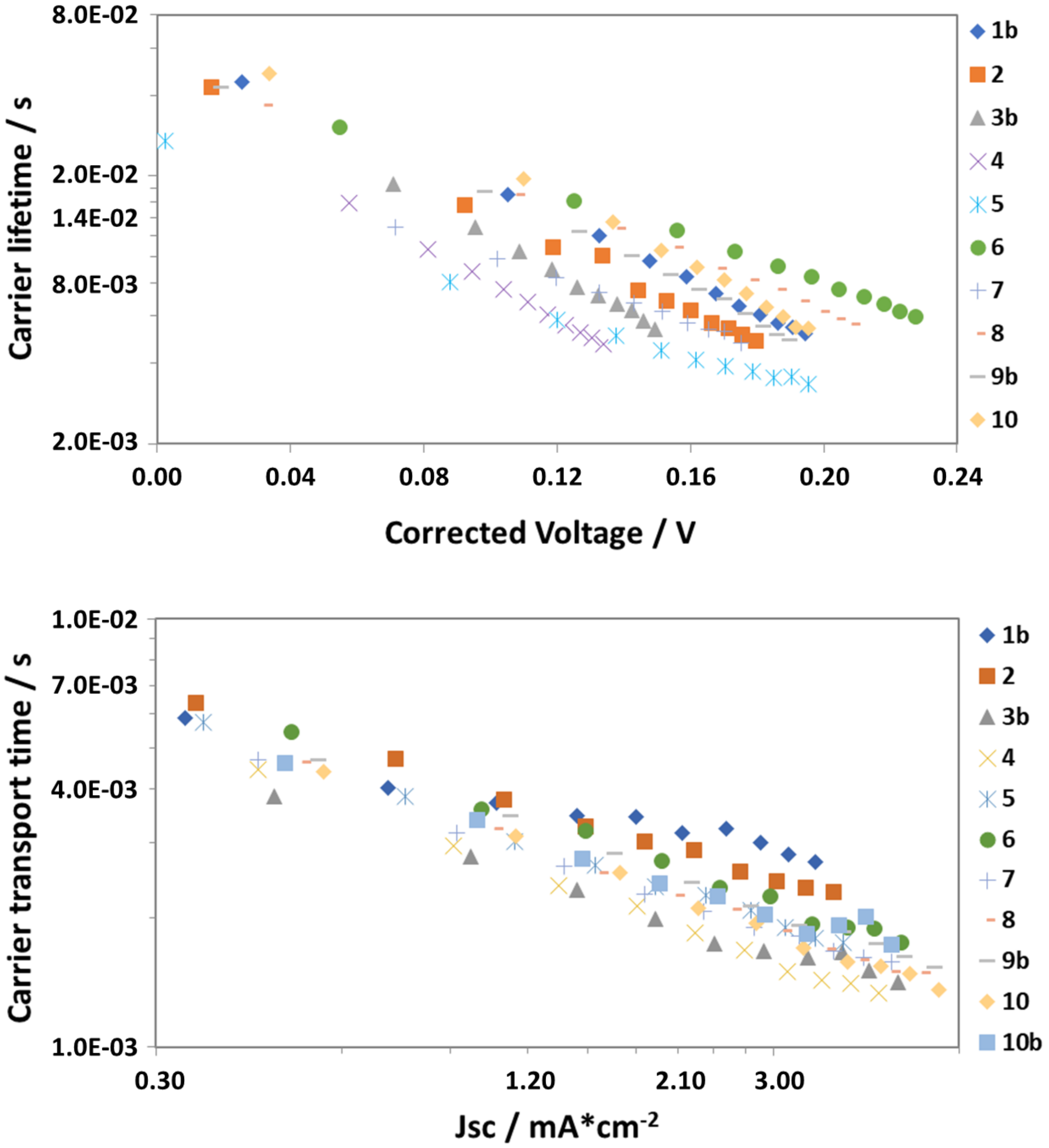

Figure S5. Electron lifetime (up) and transport time (down) as a function of light intensity. V_{OC} was corrected considering the shift of $E_{f,redox}$ due to the different redox concentrations.
